# Lifestyle-Related Risk Factors of Orthorexia Can Differ among the Students of Distinct University Courses

**DOI:** 10.3390/nu14051111

**Published:** 2022-03-06

**Authors:** Monica Guglielmetti, Ottavia Eleonora Ferraro, Ilaria Silvia Rossella Gorrasi, Elisabetta Carraro, Simona Bo, Giovanni Abbate-Daga, Anna Tagliabue, Cinzia Ferraris

**Affiliations:** 1 Human Nutrition and Eating Disorder Research Center, Department of Public Health, Experimental and Forensic Medicine, University of Pavia, 27100 Pavia, Italy; anna.tagliabue@unipv.it; 2 Unit of Biostatistics and Clinical Epidemiology, Department of Public Health, Experimental and Forensic Medicine, University of Pavia, Via Forlanini 2, 27100 Pavia, Italy; ottavia.ferraro@unipv.it; 3 Department of Public Health and Pediatrics, University of Turin, 10126 Turin, Italy; ilaria.gorrasi@unito.it (I.S.R.G.); elisabetta.carraro@unito.it (E.C.); 4 Department of Medical Sciences, University of Turin, c.so AM Dogliotti 14, 10126 Turin, Italy; simona.bo@unito.it; 5 Department of Neurosciences “Rita Levi Montalcini”, University of Turin, 10126 Turin, Italy; giovanni.abbatedaga@unito.it; 6 Laboratory of Food Education and Sport Nutrition, Department of Public Health, Experimental and Forensic Medicine, University of Pavia, 27100 Pavia, Italy

**Keywords:** orthorexia, university students, lifestyle habits, dieting, physical activity, supplements use

## Abstract

Orthorexia nervosa (ON) is defined as the excessive attention on healthy eating, and studies especially focused on food quality ON prevalence in university students can be extremely variable. The objective of this study is to investigate whether there was a difference in ON risk between health-scientific, economic-humanistic, sport sciences and dietetics and nutrition students, and to evaluate if lifestyle-related ON risk factors (dieting, physical activity, drugs and supplements use) could have an impact in different ways in determining ON risk among students attending these four programs. Participants were recruited at the University of Pavia and received a two-section questionnaire including demographic and lifestyle information and the ORTO-15 questionnaire. A total of 671 students (54% F e 46% M) completed the questionnaire (median age 21.00 (IQR 20.00–23.00), median BMI 21.77 kg/m^2^ (IQR 20.06–23.66 kg/m^2^)). The 31.2% had ORTO-15 test scores < 35, and were considered at risk of having ON. No differences were found in ON risk among the students attending the four university courses. Dieting was confirmed as the major ON risk factor for health-scientific, economic-humanistic and sport sciences students. The type of sport practiced was an important determinant of ON risk only for the economic-humanistic course, while supplements use was statistically different between sport sciences students with or without ON. Our findings may suggest that lifestyle-related risk factors of orthorexia can differ among the students of distinct university courses, but these results need to be supported by further longitudinal and prospective studies.

## 1. Introduction

Orthorexia nervosa (ON) is defined as an unhealthy obsession with eating healthy food. The term was first introduced in 1997 by the physician Steven Bratman [1], from the Greek “ortho” (meaning “straight’’ or “correct”) and “orexia,” (meaning appetite), and it indicates extreme attention on food quality rather than food quantity [2]. It is a recently identified condition; therefore, although some authors tried to clarify its diagnostic criteria [3,4,5], to date, there is no agreement on the universal definition of ON diagnostic criteria. Dunn and Bratman proposed criteria [4] which underlined the obsessive–compulsive behavior in choosing, planning, buying, preparing, and consuming/eating healthy food that individuals with ON express [4]. ON is not included in the Diagnostic and Statistical Manual of Mental Disorder of the American Psychiatric Association (DSM-5) classification and it is not currently considered an eating disorder, although it shares some psychological aspects with these conditions [6,7]. Generally, individuals with ON do not focus their attention on physical appearance or the fear of gaining weight [1]. In some cases, the fact that they associate healthy or preventive properties of illnesses to certain foods, may lead to significant dietary restrictions, and then cause malnutrition or other life-threatening medical conditions, disrupted social life and isolation [2]. ON prevalence is extremely variable worldwide and this may be influenced by various factors (i.e., the selected population or country, sociocultural differences, different test or administration method used) [8,9,10,11]. Moreover, risk factors and risk groups are currently under investigation. Data from literature suggest that sex has a contrasting role in determining ON risk: some studies found no differences in ON risk between females and males [12], while others highlighted a higher risk of ON in females [9,13,14,15,16]. Additionally, BMI shows both negative [12] and positive [17,18] associations, while dieting [9,14,15,16] and excessive physical activity [19,20,21] have been widely documented as possible risk factors. 

Data on ON prevalence in university students are contrasting: Dunn and colleagues [22] found that less than 1% of the sample fell into ON category while a 76.6% prevalence was documented by Malmborg [23] in a group of Swedish University students. It is currently unclear whether students in health-oriented academic programs, highly focused on nutrition and physical exercise, are more prone to develop orthorexia nervosa than students in other educational areas. Bo and colleagues [24] suggested that university courses that lead to a high level of information on nutrition and healthcare could attract students with increased sensitivity to these issues. Moreover, in recent years, some studies revealed a higher risk of developing ON in students attending courses related to health, nutrition or sports [23,25,26]. Gorrasi and colleagues [13], evaluating 918 students from different Italian Universities, found that 29% of the sample expressed traits of ON, highlighting relations with age, sex, supplements use and dieting. Dell’Osso et al. [27] conducted a survey among 2130 students at the University of Pisa, demonstrating that more than one-third of the sample showed ON symptoms (ORTO-15 < 35), with higher rates among females. Diet type predicted ON and ORTO-15 total score more than sex [27]. A cross-sectional study [28], using ORTO 11 test in a sample of 534 Spanish university students, found that 30.5% of the sample were at high risk of developing ON. Higher orthorexic rates were observed in women. Those at high risk of ON showed higher BMI, a higher proportion of veganism/vegetarianism and significantly higher scores on the Multidimensional Body Shape Relations Questionnaire (MBSRQ-45) and Eating Attitudes Test (EAT-26) tests, evaluating self-image, concern with physical appearance and Eating Disorders (ED) behaviors. One study [29] compared ON prevalence between 116 student athletes and 99 non-athlete controls from Universities in the North- East of the UK completing the ORTO-15 test (≤40 cutoff). ON symptoms were high in all students (76%), while there was no difference in ORTO-15 scores between the groups. On the contrary, there was a difference in scores between those who completed ≥10 h of exercise per week and those who did ≤10 h a week. ORTO-15 scores were not higher in athletes competing in aesthetic- and weight-dependent sports. According to these results, being a student athlete for University sports team did not affect ON risk; however, it appears to be a greater risk for students in general, and for athletes who undertake high volumes of exercise. Recently, some studies [30,31,32,33,34] focused on ON risk in dietetics and nutrition students. This group may be considered at a higher risk of orthorexia due to the higher level of awareness and information on healthy eating. Caferoglu and Toklu [33] reported a higher prevalence of orthorexia in dietetic students (63.8%) than dietitians (52.9%), inversely correlated to graduation and positively associated with eating disorders. In fact, the authors found that the subscales of EAT-26 (dieting, bulimia, and oral control) were associated with higher ON tendency in these students. Grammatikopoulou and colleagues [34], evaluating 215 Greek nutrition and dietetics undergraduates, found a relationship between ON risk and reduced energy intake that can be ascribed to dieting.

So, different studies looked into ON risk factors among university students, but few studies focused on the possible different impacts of lifestyle-related ON risk factors in students attending specific university courses. Thus, we decided to investigate whether there was a difference in ON risk between health-scientific, economic-humanistic, sport sciences and dietetics and nutrition students. Then, we wanted to evaluate if lifestyle-related ON risk factors (dieting, physical activity, drugs and supplements use) could impact in different ways in determining ON risk among students attending these four programs. We hypothesize that nutrition and sport sciences students could have a higher risk of orthorexia compared to the other under-graduates. This could be linked to the higher level of awareness and to the higher level of physical activity (specifically sport) practiced, respectively.

## 2. Materials and Methods

### 2.1. Study Design

This was an observational cross-sectional study conducted by the Human Nutrition and Eating Disorder Research Center of the University of Pavia.

### 2.2. Participants

Participants were recruited from among University of Pavia students. Students were approached by an expert dietician during their habitual university lessons, with the proposal to complete a paper survey evaluating nutritional habits and physical activity levels among undergraduates. No references were made to the purpose of investigating ON risk among students. Participants were invited to anonymously fill in a two-section questionnaire, without any kind of remuneration or credits for their curricula. The study procedures were clearly explained to the students and written informed consent was obtained for those who decided to participate in the study.

#### 2.2.1. Inclusion and Exclusion Criteria

To participate in the survey student had to comply with the following criteria: to be attending the first year of a university course belonging to the four areas of study as follows, according to our previous study [13]:

1. health-scientific: biology, speech therapy, medicine, optics and optometry, physiotherapy, childhood neuro-psychomotricity therapy, psychology
2. economic-humanistic: business administration, economy and management, law, political and social sciences
3. sport sciences: exercise and sport sciences
4. dietetics and nutrition

Students who did not meet the previous criteria or did not completely fill out the questionnaire were excluded from the study.

#### 2.2.2. Questionnaire

A two-section questionnaire was administered: the first section included demographic and lifestyle information, while the second section was completely composed of the ORTO-15 questionnaire.

The first section of the questionnaire included personal information, physical activity, drugs and supplements use and dietary habits information. Participants had to report their age, sex (male/female), body weight (in kilograms), height (in meters) and university curriculum. Body Mass Index was calculated on the basis of reported weight and height. Physical activity was investigated both in terms of frequency (number of times per week and number of hours per week) and type of exercise. Students had to choose and then indicate their habitual physical activity (in terms of sport done) between running, swimming, cycling, football, aerobic exercises (gym), weight lifting (gym), dance, volleyball, athletics or other (specify). In the case of students who practiced multiple sports, more than one answer was admitted.

As regards drugs and supplements, the questionnaire asked “Do you use dietary supplements?” and “Do you use prescribed medications?”. Participants had to answer Yes/No and, if yes, to specify the type of dietary supplement or prescribed medication used.

The last question of this part wanted to investigate whether students followed a diet or not. Those who declared themselves to be dieting had to specify the type of diet followed (for example vegetarian/vegan diet, low-carbohydrates diet etc).

The second section of the questionnaire was composed of the ORTO-15 test, developed and validated for the Italian population by Donini and colleagues [35]. It is composed of 15 multiple-choice items and it investigates the obsessive attitude of people in choosing, buying, preparing and consuming food that they consider healthy. Items are divided into cognitive-rational, clinical and emotional categories. The answers are based on a four-point Likert scale (always, often, sometimes, never), with a score from 1 to 4, where the lowest values of the score indicate a greater risk for ON. There are two possible cutoffs (<35 and <40). In this investigation we calculated ON risk considering both 35 and 40 as cutoff. As suggested by Donini et al. [30] the <40 threshold has a high sensitivity, while the <35 cut-off has a greater specificity (94.2%) and negative predictive value (91.1%). So, we utilized <35 threshold for the comparisons and analysis.

#### 2.2.3. Variables

After recruitment and questionnaire completion, students were coded into four major “curricula classes” (A, B, C, D), according to the university courses attended. Those who followed health-scientific courses were included in Class A, students of economic-humanistic courses were inserted in Class B. Sport sciences students were encoded in Class C, while dietetics and nutrition students were in Class D.

Physical activity was reported as minutes of sport practiced per week and in six classes considering the type of sport practiced (aerobic sports, strength sports, aesthetic sports, combat sports, team sports and multiple sports). Aerobic activities included running, swimming, cycling, athletics and aerobic exercises (gym). Weight lifting and crossfit were considered a strength sport while dance, pole dance, horse riding, rhythmic and artistic gymnastics were collected in aesthetic sports. Martial arts, taekwondo, judo, karate, kickboxing, were collected in combat sports. Football, basketball, rugby and volleyball were classified as team sports and the category “multiple sport” included those who practiced more than one sport.

For prescribed medications, dietary supplements and dieting a binomial (Yes/No) classification was used. On the basis of the answers given from the students, further groupings were done both for dietary supplements used (muscle gain supplements as whey protein and amino acids or multivitamins or mineral salts) and for types of diet followed (hypo-energetic diets, hyper-energetic-high-protein diets, low-carbohydrates diet, low-fat diet, vegetarian/vegan and/or therapeutic diets such as gluten-free diet and lactose-free diets).

### 2.3. Ethical Approval

This research was reviewed and approved by the Bioethical Committee of the University of Turin on 29 January 2014. Everyone provided written consent to participate in the present study. The study was carried out according to the Declaration of Helsinki.

### 2.4. Statistical Analysis

Quantitative variables were summarized as mean and standard deviations (SD) or medians and interquartile ranges (IQR), the qualitative variables were summarized as percentages. The normality of distribution was assessed with the Shapiro-Wilk test. To explore the differences between groups for quantitative variables, an Anova or t-test or the analogous nonparametric tests were implemented; the chi square test was used to compare qualitative factors instead. When appropriate, differences between pair groups were tested and the Bonferroni correction was applied for multiple comparisons. To evaluate possible associations between the risk of ON and demographic and lifestyle variables collected, a multivariable logistic regression model was performed in each university group and odds ratios (OR) with 95% confidence interval (95% CI) were reported. A *p*-value less than 0.05 was considered significant (two-sided). All the analyses were performed using STATA^®^ 17 [36].

## 3. Results

### 3.1. Sample Characteristics

At the beginning of enrollment, 689 students decided to participate, but 18 participants did not complete the questionnaire (they did not differ from statistical point of view in terms of age and sex. Data not shown). The main characteristics (age, BMI, sex, minutes of sport/week, type of sport, ORTO-15 < 35, dieting and supplements use) both of the entire sample and of the students meeting the four university curricula evaluated are reported in Table 1.

The final sample was composed of 671 participants with 54% of females and 46% males. Median age was 21.00 (IQR 20.00–23.00), median BMI 21.77 kg/m^2^ (IQR 20.06–23.66 kg/m^2^). The 9.5% (*n* = 64) were underweight while 13.3% (*n* = 89) were overweight.

In total, 169 (25.2%) participants attended health-scientific courses, 192 students (28.6%) studied economic-humanistic programs, 218 participants studied sport sciences (32.5%), and the remaining 92 studied dietetics and nutrition (13.7%).

### 3.2. Diet, Supplements, Physical Activity, and Orthorexia Risk

In the whole sample, 31.2% (*n* = 209) students had ORTO-15 test scores < 35, so they were considered at risk of having ON. No statistically significant difference was found between participants at or without ON risk, both for sex (*p* = 0.587) and BMI (*p* = 0.168). Moreover, no statistical difference was found in ON risk among the students attending the four university courses considered (*p* > 0.90). In fact, the percentage of participants with ON risk was about 30% in all the university courses evaluated.

Only 8.6% of the sample reported not doing any kind of physical activity or sport, while a median of 240 min/week (IQR 120.00–480.00) was reported. Fifty-nine different types of sports were reported and then classified in the following categories: aerobic sports, strength sports, aesthetic sports, team sports, combat sports and multiple sports. Among participants, 27.7% of them practiced aerobic sports, 11.9% did strength sports, 5.5% aesthetic sports, 10.9% team sports, 2.7% combat sports and 32.6% multiple sports. As regards dieting, 11.6% of the sample (*n* = 78) stated that they were following a diet: more precisely 30.8% hypo-energetic diets, 23.1% hyper-energetic-high-protein diets, 12.8% were vegetarians/vegans and 33.3% followed other type of diets (e.g., gluten-free diet, lactose-free diet). Considering supplements use itself, 18.3% (*n* = 123) of the participants declared to take them, similarly for drugs with 12.8%. The majority of the participants who declared taking medications were females, who stated the use of the contraceptive pill. Whey protein, amino acids, multivitamins and mineral salts were the main dietary supplements utilized.

Nutrition students had a higher age (23 years as median; *p* < 0.001) and a higher prevalence of females (73.91%, *p* < 0.001) in comparison with the other groups. As expected, the higher median as minutes of physical activity was found in students of sport science (480 min, *p* < 0.001). Aerobic sports were the most practiced by students of nutrition and economic-humanistic courses, while in the other two curricula multiple sports were mostly practiced (*p* < 0.001) (Table 1). No statistical differences were found for the types of diet followed (*p* = 0.873 data not shown).

### 3.3. University Curricula and ON Lifestyle-Related Risk Factors

A detailed description of the characteristics of the health-scientific, economic-humanistic, sport sciences, dietetics and nutrition students is presented in Table 2, respectively.

In health-scientific students we found a statistically significant difference among students with/without ON risk as regards dieting. In fact, the odds of being at risk of ON was 3.14 [CI 1.16–8.49] (*p* < 0.019) times higher among those who indicated to follow a diet in comparison to the participants who did not for the best model found for this group. Moreover, the number of students who declared dieting more than doubled those without ON risk (18.87% vs. 6.90%) (Table 2).

Similarly, in the best model for economic-humanistic students (Table 2) the risk of ON in those who indicated following a diet in comparison to those who did not was 5.04 [CI 1.85–13.69] (*p* = 0.002) times higher. Moreover, the odds of being at risk of ON was lower for the participants who practiced aerobic sports in comparison to those who declared to do multiple sports (OR = 0.32 [CI 0.13–0.81]) (*p* = 0.016).

Among sport sciences students, the best logistic model showed that the odds of being at risk of ON was 3.04 times higher in those who declared following a diet in comparison to those who did not. From Table 2 it is possible to see that only the 7.48% of sport sciences students without risk of ON indicated following a diet, in contrast with the 19.72% of students at risk of ON (*p =* 0.008). Moreover, we found a statistically significant difference between the students with/without ON risk as regards supplements use. In fact, those who stated to take supplements and were at risk of ON doubled those who were not at risk of ON and declared to take supplements (29.58% vs. 14.97%).

In dietetics and nutrition students we found a statistically significant difference as regards minutes of sport per week practiced (Table 2, *p* = 0.014), but this evidence was not confirmed by logistic regression analysis. Finally, for all the four subgroups analyzed, the type of diet followed was not statistically significant (data not shown). So, for each subgroup, there were no differences between students with and without ON risk on the only basis of the type of diet followed.

## 4. Discussion

In our study we found a percentage of subjects at risk of ON similar to those reported in previous studies on Italian University students [13,24,27]. Our results confirm dieting as one of the major determinants in orthorexia nervosa risk and introduce a new perspective on the different impact of each lifestyle-related factor in conditioning ON risk among students attending different university programs. In fact, on the basis of our findings, dieting, physical activity, drugs and supplements use, act in different ways on the probability of students developing ON risk.

As revealed from various studies [23,25,27], university curricula can influence ON risk, especially for those who choose nutrition, sport or health-related courses. In contrast with our hypothesis and with these findings, in our study no differences were found in ON risk among the students attending the four university courses considered. Instead, we highlighted a difference in the specific factor that determined ON risk in each university course. Dieting was the only determinant in ON risk among students attending health-scientific programs. This was also confirmed in economic-humanistic undergraduates, in which the type of sport practiced seemed to be a “protective” factor. Sport sciences students seemed to be more prone to developing orthorexia if they followed a diet and used supplements, while the intensity and type of sport practiced was not correlated to ON risk. At the same time, in dietetics and nutrition students we found a statistically significant difference as regards minutes of sport per week practiced, but this evidence was not confirmed by logistic regression analysis. Therefore, there are important differences between the various types of factors in determining ON risk.

Moreover, our study confirmed the importance of dieting as an ON risk factor. Previous studies suggested a relationship between ON and specific, restrictive nutritional approaches, from simply being on a diet to adhering to vegetarianism and other specific dietary approaches [9,13,14,15,37,38,39]. Although in this study we did not find any relationship with specific diet regimes, probably because of the reduced number of participants who specified the type of diet followed, data showed that the diet played an essential role. This can be determined by different factors: from one side, ON behaviors may be conditioned by the “obsessive” tendency to following a healthy diet and to the consequent restriction of food consumption. On the other side, ON can be a consequence of weight loss or body composition modeling approaches that can influence food choices. Different characteristics referring to dietetic (restrictive, characterized by the avoidance of certain foods, distorted eating habits, shortage of essential nutrients, leading to malnutrition and underweight) or behavioral aspects (ritualized, strictly controlled, becoming the central focus of life, with modification of social relationships) should be taken into account [40]. The fact that nutrition and dietetics students are aware of this relationship could be the explanation of the fact that we did not find any correlation between dieting and ON risk.

The use of concentrated food supplements is present in the proposed diagnostic criteria for ON by Dunn and Bratman as well as dietary practice [4,13]. In our study we reported a significant difference in supplements use between sport sciences students with or without ON risk. These results may be explained considering the type of supplement predominantly used (amino acids, proteins, multivitamin and mineral salts) that are frequent for those who practice physical activity, especially strength sports, and often are on a restrictive diet. In fact, those students also had a higher rate of dieting. In our opinion, there is a relationship between supplements use and dieting that should be further investigated.

These results led to hypothesize the role of general lifestyle habits in determining ON risk. Due to the cross-sectional design of the study, it is not possible to define whether lifestyle habits conditioned ON risk or, on the contrary, if ON symptoms tendency influenced everyday habits of these students.

Our study presents some strengths and limitations. Firstly, we evaluated a quite large number of students, of different ages and courses. Secondly, we studied the relationship not only with physical activity itself but also with specific types of sports and minutes of sport practiced. One of the limitations is that in our sample we did not investigate the presence/absence of other eating disorders that are known to be linked to nervous orthorexia risk. We included only students attending the first year of university, so our results may not be generalized to all the students of the specific courses. Moreover, in evaluating the lifestyle of our participant, we did not investigate smoking habits and sleep quality. Then, according to the recent research [30], the ORTO-15 test is questionable due to a high percentage of falsely positive results, so it cannot surely distinguish between orthorexia nervosa and healthy eating. Last, the survey collected only self-reported data.

## 5. Conclusions

In conclusion, our findings may suggest that lifestyle-related risk factors of orthorexia can differ among the students of distinct university courses, but these results need to be supported by further longitudinal and prospective studies. Nevertheless, screening among university students is not only desirable but essential, in order to have an early identification of students at risk, and then set up adequate prevention and treatment.

## Figures and Tables

**Table 1:** Main characteristics (age, BMI, sex, minutes of sport/week, type of sport, ORTO-15 cut off, dieting and supplements use) of the entire sample and of the students attending the 4 university curricula evaluated.

Characteristics		Total	Health-Scientific	Economic-Humanistic	Sport Sciences	Dietetics and Nutrion	*p*-Value
		*n* = 671	*n* = 169	*n* = 192	*n* = 218	*n* = 92	
Age, median (IQR)		21.00 (20.00–23.00)	21.00 (20.00–23.00)	21.00 (20.00–22.00)	21.00 (20.00–23.00)	23.00 (20.50–26.00)	<0.001
BMI, median (IQR)		21.77 (20.06–23.66)	21.60 (19.49–23.66)	21.47 (19.72–23.52)	22.12 (20.45–23.88)	21.94 (20.40–23.77)	0.083
Sex, *n* (%)	F	362 (53.95%)	89 (52.66%)	117 (60.94%)	88 (40.37%)	68 (73.91%)	<0.001
	M	309 (46.05%)	80 (47.34%)	75 (39.06%)	130 (59.63%)	24 (26.09%)	
Minutes/ week, median (IQR)		240.00 (120.00–480.00)	180.00 (120.00–360.00)	180.00 (60.00–300.00)	480.00 (240.00–720.00)	240.00 (120.00–360.00)	<0.001 #
Type of sport *n* (%)	No sports	58 (8.64%)	17 (10.06%)	35 (18.23%)	0 (0.00%)	6 (6.52%)	<0.001
	Aerobic sports	186 (27.72%)	45 (26.63%)	63 (32.81%)	32 (14.68%)	46 (50.00%)	
	Strength sports	80 (11.92%)	16 (9.47%)	23 (11.98%)	31 (14.22%)	10 (10.87%)	
	Aesthetic sports	37 (5.51%)	12 (7.10%)	7 (3.65%)	10 (4.59%)	8 (8.70%)	
	Combat sports	18 (2.68%)	3 (1.78%)	4 (2.08%)	9 (4.13%)	2 (2.17%)	
	Team sports	73 (10.88%)	15 (8.88%)	16 (8.33%)	41 (18.81%)	1 (1.09%)	
	Multiple sport	219 (32.64%)	61 (36.09%)	44 (22.92%)	95 (43.58%)	19 (20.65%)	
ORTO-15 < 35	No	462 (68.85%)	116 (68.64%)	135 (70.31%)	147 (67.43%)	64 (69.57%)	0.936
	Yes	209 (31.15%)	53 (31.36%)	57 (29.69%)	71 (32.57%)	28 (30.43%)	
Dieting, *n* (%)	No	593 (88.38%)	151 (89.35%)	171 (89.06%)	193 (88.53%)	78 (84.78%)	0.704
	Yes	78 (11.62%)	18 (10.65%)	21 (10.94%)	25 (11.47%)	14 (15.22%)	
Drugs, *n* (%)	No	86 (12.82%)	16 (9.47%)	35 (18.23%)	16 (7.34%)	19 (20.65%)	<0.001
	Yes	585 (87.18%)	153 (90.53%)	157 (81.77%)	202 (92.66%)	73 (79.35%)	
Supplements use, *n* (%)	No	548 (81.67%)	136 (80.47%)	166 (86.46%)	175 (80.28%)	71 (77.17%)	0.201
	Yes	123 (18.33%)	33 (19.53%)	26 (13.54%)	43 (19.72%)	21 (22.83%)	

BMI = Body Mass Index. All the post-hoc tests were statistically significant except for contrast between health-scientific and sport science students. # All the post-hoc tests were statistically significant except for contrast between health-scientific and nutrition students. Data are presented as median (IQR) for continuous measures, and *n* (%) for categorical measures.

**Table 2:** (**a**) Comparison between the health-scientific students with or without ON risk. (**b**) Comparison between economic-humanistic students with or without ON risk. (**c**) Comparison between sport sciences students with or without ON risk. (**d**) Comparison between dietetics and nutrition students with or without ON risk.

(**a**)					
**Health-Scientific**		**Total**	**Without ON Risk**	**With ON Risk**	***p*-Value**
		***n* = 169**	***n* = 116**	***n* = 53**	
Age, median(IQR)		21.00 (20.00–23.00)	21.00 (20.00–23.00)	21.00 (20.00–22.00)	0.199
BMI, median(IQR)		21.60 (19.49–23.66)	21.68 (19.40–23.77)	21.22 (19.83–23.34)	0.947
Sex, *n* (%)	F	89 (52.66%)	62 (53.45%)	27 (50.94%)	0.762
	M	80 (47.34%)	54 (46.55%)	26 (49.06%)	
Minutes/week, median(IQR)		180.00 (120.00–360.00)	180.00 (120.00–360.00)	240.00 (120.00–360.00)	0.105
Type of sport *n* (%)	No sports	17 (10.06%)	14 (12.07%)	3 (5.66%)	0.044
	Aerobic sports	45 (26.63%)	33 (28.45%)	12 (22.64%)	
	Strength sports	16 (9.47%)	11 (9.48%)	5 (9.43%)	
	Aesthetic sports	12 (7.10%)	9 (7.76%)	3 (5.66%)	
	Combat sports	3 (1.78%)	2 (1.72%)	1 (1.89%)	
	Team sports	15 (8.88%)	14 (12.07%)	1 (1.89%)	
	Multiple sport	61 (36.09%)	33 (28.45%)	28 (52.83%)	
Dieting, *n* (%)	No	151 (89.35%)	108 (93.10%)	43 (81.13%)	0.019
	Yes	18 (10.65%)	8 (6.90%)	10 (18.87%)	
Drugs, *n* (%)	No	16 (9.47%)	10 (8.62%)	6 (11.32%)	0.578
	Yes	153 (90.53%)	106 (91.38%)	47 (88.68%)	
Supplements use, *n* (%)	No	136 (80.47%)	93 (80.17%)	43 (81.13%)	0.883
	Yes	33 (19.53%)	23 (19.83%)	10 (18.87%)	
(**b**)					
**Economic-Humanistic**		**Total**	**Without ON Risk**	**With ON Risk**	***p*-Value**
		***n* = 192**	***n* = 135**	***n* = 57**	
Age, median(IQR)		21.00 (20.00–22.00)	20.00 (19.00–22.00)	21.00 (20.00–22.00)	0.803
BMI, median(IQR)		21.47 (19.72–23.52)	21.48 (19.88–23.44)	21.30 (19.38–23.55)	0.842
Sex, *n* (%)	F	117 (60.94%)	78 (57.78%)	39 (68.42%)	0.167
	M	75 (39.06%)	57 (42.22%)	18 (31.58%)	
Minutes/week, median(IQR)		180.00 (60.00–300.00)	120.00 (60.00–240.00)	240.00 (78.00–360.00)	0.111
Type of sport *n* (%)	No sports	35 (18.23%)	23 (17.04%)	12 (21.05%)	0.035
	Aerobic sports	63 (32.81%)	52 (38.52%)	11 (19.30%)	
	Strength sports	23 (11.98%)	13 (9.63%)	10 (17.54%)	
	Aesthetic sports	7 (3.65%)	4 (2.96%)	3 (5.26%)	
	Combat sports	4 (2.08%)	2 (1.48%)	2 (3.51%)	
	Team sports	16 (8.33%)	14 (10.37%)	2 (3.51%)	
	Multiple sport	44 (22.92%)	27 (20.00%)	17 (29.82%)	
Dieting, *n* (%)	No	171 (89.06%)	127 (94.07%)	44 (77.19%)	<0.001
	Yes	21 (10.94%)	8 (5.93%)	13 (22.81%)	
Drugs, *n* (%)	No	35 (18.23%)	22 (16.30%)	13 (22.81%)	0.286
	Yes	157 (81.77%)	113 (83.70%)	44 (77.19%)	
Supplements use, *n* (%)	No	166 (86.46%)	121 (89.63%)	45 (78.95%)	0.048
	Yes	26 (13.54%)	14 (10.37%)	12 (21.05%)	
(**c**)					
**Sport Sciences**		**Total**	**Without ON Risk**	**With ON Risk**	***p*-Value**
		***n* = 218**	***n* = 147**	***n* = 71**	
Age, median (IQR)		21.00 (20.00–23.00)	21.00 (20.00–23.00)	22.00 (20.00–23.00)	0.041
BMI, median (IQR)		22.12 (20.45–23.88)	21.91 (20.43–23.81)	22.49 (20.57–24.15)	0.214
Sex, *n* (%)	F	88 (40.37%)	59 (40.14%)	29 (40.85%)	0.920
	M	130 (59.63%)	88 (59.86%)	42 (59.15%)	
Minutes/week, median (IQR)		480.00 (240.00–720.00)	480.00 (300.00–720.00)	420.00 (180.00–720.00)	0.200
Type of sport *n* (%)	Aerobic sports	32 (14.68%)	21 (14.29%)	11 (15.49%)	0.209
	Strength sports	31 (14.22%)	18 (12.24%)	13 (18.31%)	
	Aesthetic sports	10 (4.59%)	7 (4.76%)	3 (4.23%)	
	Combat sports	9 (4.13%)	8 (5.44%)	1 (1.41%)	
	Team sports	41 (18.81%)	33 (22.45%)	8 (11.27%)	
	Multiple sport	95 (43.58%)	60 (40.82%)	35 (49.30%)	
Dieting, *n* (%)	No	193 (88.53%)	136 (92.52%)	57 (80.28%)	0.008
	Yes	25 (11.47%)	11 (7.48%)	14 (19.72%)	
Drugs, *n* (%)	No	16 (7.34%)	6 (4.08%)	10 (14.08%)	0.008
	Yes	202 (92.66%)	141 (95.92%)	61 (85.92%)	
Supplements use, *n* (%)	No	175 (80.28%)	125 (85.03%)	50 (70.42%)	0.011
	Yes	43 (19.72%)	22 (14.97%)	21 (29.58%)	
(**d**)					
**Dietetics and Nutrition**		**Total**	**Without ON Risk**	**With ON Risk**	***p*-Value**
		***n* = 92**	***n* = 64**	***n* = 28**	
Age, median(IQR)		23.00 (20.50–26.00)	23.00 (21.00–26.00)	22.50 (20.00–25.50)	0.338
BMI, median(IQR)		21.94 (20.40–23.77)	21.62 (20.28–23.45)	22.49 (20.65–24.01)	0.268
Sex, *n* (%)	F	68 (73.91%)	47 (73.44%)	21 (75.00%)	0.875
	M	24 (26.09%)	17 (26.56%)	7 (25.00%)	
Minutes/week, median (IQR)		240.00 (120.00–360.00)	180.00 (120.00–300.00)	300.00 (180.00–420.00)	0.014
Type of sport, *n* (%)	No sports	6 (6.52%)	6 (9.38%)	0 (0.00%)	0.379
	Aerobic sports	46 (50.00%)	31 (48.44%)	15 (53.57%)	
	Strength sports	10 (10.87%)	8 (12.50%)	2 (7.14%)	
	Aesthetic sports	8 (8.70%)	6 (9.38%)	2 (7.14%)	
	Combat sports	2 (2.17%)	1 (1.56%)	1 (3.57%)	
	Team sports	1 (1.09%)	0 (0.00%)	1 (3.57%)	
	Multiple sport	19 (20.65%)	12 (18.75%)	7 (25.00%)	
Dieting, *n* (%)	No	78 (84.78%)	55 (85.94%)	23 (82.14%)	0.753
	Yes	14 (15.22%)	9 (14.06%)	5 (17.86%)	
Drugs, *n* (%)	No	19 (20.65%)	13 (20.31%)	6 (21.43%)	0.903
	Yes	73 (79.35%)	51 (79.69%)	22 (78.57%)	
Supplements use, *n* (%)	No	71 (77.17%)	50 (78.13%)	21 (75.00%)	0.742
	Yes	21 (22.83%)	14 (21.88%)	7 (25.00%)	

BMI = Body Mass Index. Data are presented as median (IQR) for continuous measures, and *n* (%) for categorical measures.

## Data Availability

The data presented in this study are available on request from the corresponding author. The data are not publicly available due to privacy reasons.
